# Refractory hypokalemia with sexual dysplasia and infertility caused by 17α-hydroxylase deficiency and triple X syndrome: A case report

**DOI:** 10.1515/biol-2022-0548

**Published:** 2023-02-13

**Authors:** Jun-Teng Yao, Ming-Zhi Xu, Yu-Ren Zhang, Bai-Rong Wang, Mei-Rong Li, Lu Gao

**Affiliations:** Department of Endocrinology, Jinjiang Municipal Hospital, No. 16 of Luoshan section, Jinguang Road, Jinjiang 362200, China; Department of Endocrinology, Shulan Hospital, Hangzhou 310000, China; Department of Endocrinology, Jinjiang Municipal Hospital, Jinjiang 362200, China

**Keywords:** congenital adrenal hyperplasia, 17α-hydroxylase deficiency, CYP17A1 gene abnormality, refractory hypokalemia, triple X syndrome, sexual hypoplasia, infertility

## Abstract

The present study reports a patient case with a 17α-hydroxylase deficiency accompanied by triple X syndrome. A 17α-hydroxylase deficiency leads to a very low 17α-hydroxylated steroid synthesis as well as a non-feedback increase in the adrenocorticotropic hormone level. Meanwhile, the progesterone level increases the 17α-hydroxyprogesterone level and decreases the dehydroepiandrosterone sulfate level. The patient is characterized by intractable hypokalemia, high urinary potassium, hyperaldosteronemia, hyporeninemia, hypocortisolemia, hypertension, gonadal and secondary sexual dysplasia, a decreased estrogen level, primary amenorrhea, and infertility. The imaging findings indicate a presence of multiple bilateral adrenal gland adenomas, and the sequencing indicates a missense CYP17A1-E7 gene pathogenic variant. The karyotype is a 47, XXX [3]/46, XX [47] low-level chimeric karyotype. The patient’s parents are cousins. To our knowledge, this patient is the first case diagnosed with congenital adrenal hyperplasia caused by hydroxylase deficiency and triple X syndrome. The uniqueness of this case is that this patient has two very rare genetic diseases, probably due to the marriage of close relatives.

## 1 Background

A 17α-hydroxylase deficiency is caused by a CYP17 gene pathogenic variant that encodes the enzyme; the CYP17A1 gene was cloned from a human testicular tissue cDNA library in 1987 [1]. The gene encoding CYP17 is located in the long arm of chromosome 10 (10q24–25), which contains 11 exons and 10 introns and has a length of approximately 13 kb [1]. In recent years, over 50 types of CYP17 gene pathogenic variant have been reported [2], and new pathogenic variant types are continuously being found [3,4]. The CYP17 gene is involved in steroid biosynthesis in both the adrenal gland and the gonad; therefore, the enzyme deficiency results in an extremely low 17α-hydroxylated steroid (androgen, estrogen, cortisol, 11-deoxycortisol, and 17-OPH) synthesis, along with a non-feedback adrenocorticotropic hormone (ACTH) increase [5]. Meanwhile, the progesterone level increases, and the 17α-hydroxyprogesterone and dehydroepiandrosterone sulfate levels decrease [5]. A previous study summarized the gene pathogenic variant status of 181 Chinese patients with 17α-hydroxylase deficiency and found that 70 (38.6%) with c.985_987delinsAA (p.Y329K) pathogenic variant, 55 (30.4%) with c.1459-1467del (p.487_489del) pathogenic variant, and 43 (31%) with other pathogenic variants [6]. In patients with 17α-hydroxylase deficiency, inhibition of the synthesis of cortisol and sex hormones leads to increased secretion of 11-deoxycorticosterone, resulting in hyperaldosteronism, clinical hypokalemia alkalosis, and hypertension [6].

The 47, triple X syndrome is a sex chromosome aneuploidy (SCA) disorder with an extra X sex chromosome compared to the normal female karyotype 46,XX. In 1959, Jacobs et al. first reported a 35 year old woman with normal intelligence diagnosed after 6 years of secondary amenorrhea [7]. Since then, hundreds of women have been reported for developmental delays, psychological disorders, or other different manifestations [8,9]. According to recent data from the Danish Cytogenetic Registry, the incidence of the 47,XXX syndrome is about 11/10,000 in women, and only nearly 13% of patients are diagnosed [10], which is similar to the previously reported incidence of 1 in 1,000 and a confirmation rate of 10% based on newborn screening [11].

Here we report a case of 17α-hydroxylase deficiency combined with triple X syndrome, and the clinical manifestations, physical signs, supplementary examinations, and genetic characteristics were analyzed.


**Informed consent:** Informed consent has been obtained from all individuals included in this study.
**Ethical approval:** The research related to human use has been complied with all the relevant national regulations, institutional policies and in accordance with the tenets of the Helsinki Declaration, and has been approved by the authors’ institutional review board or equivalent committee.

## 2 Case presentation

The patient is a 56 year old female farmer admitted to the Department of Endocrinology with the chief complaint of limb fatigue for over 10 years (aggravated for 3 days). The patient had a history of hypertension for 10 years and was treated with oral antihypertensive drugs for 1 year (details unavailable). Her blood pressure (BP) was not monitored. The patient was married at the age of 25 and has a normal sexual life; however, she has not experienced pregnancy or childbearing (0-0-0-0). Furthermore, the patient has experienced no instance of menstruation since birth. This patient visited the local clinic when she was young due to primary amenorrhea but did not undergo examinations and treatments. She had no history of surgery. We have added this information in Section 1.

### 2.1 Family details

**Figure 1:**
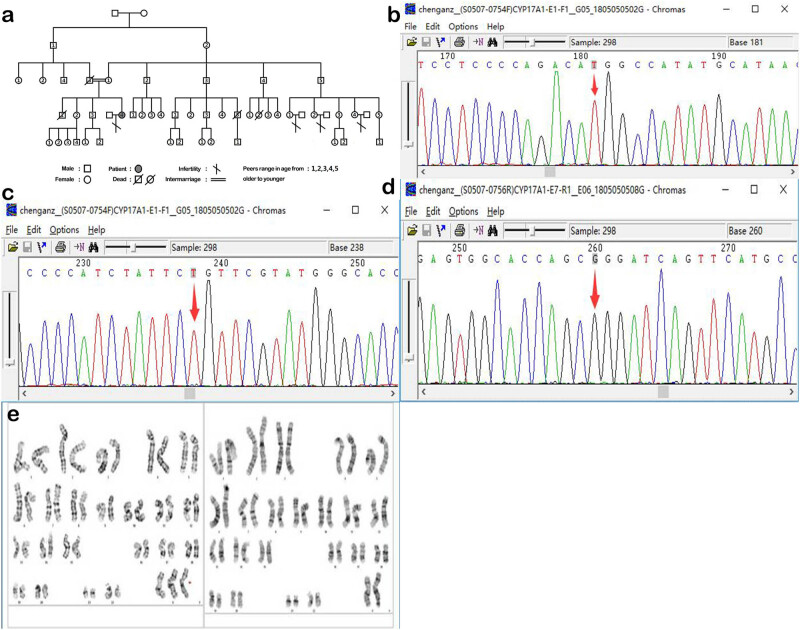
(a) Family diagram of patients; (b) CYP17A1 gene sequencing 1; genetic testing revealed that the cytosine (nucleotide C) at the site 181 of the 17α-hydroxylase-related CYP17 gene underwent a synonymous mutation and was changed to thymine (nucleotide T); and (c) CYP17A1 gene sequencing 2; genetic testing revealed that the guanine (nucleotide G) at the site 238 of the 17α-hydroxylase-related CYP17 gene underwent a synonymous mutation and was changed to thymine (nucleotide T); (d) CYP17A1 gene sequencing 3; genetic testing revealed that the cytosine (nucleotide C) at the site 1226 of the 17α-hydroxylase-related CYP17 gene underwent a missense mutation and was changed to guanine (nucleotide G); (e) karyotype.

The patients’ parents are cousins. She had an older brother who died at a young age (details unavailable) and has many female relatives (cousins) who are married and have no children. The family tree is shown in Figure 1a.

### 2.2 Physical examination

Special physical examination at admission: BP = 142/92 mmHg, height = 168 cm, body weight = 60 kg, and body mass index = 23.4 kg/m². The patient’s intelligence was normal, there was no abnormal skin pigmentation, the ocular fissure was not widened, and the thyroid gland was not enlarged. The patient had no axillary hair or pubic hair (Tanner stage P1). The breast size was normal, the papillae was at Tanner stage B5, the breast distance was widened (27 cm), the areola was light brown in color, the external genitalia presented with hypoplasia (the labia major, labia minor, and clitoris were infantile), and no deformity or clubbing finger was found in the patient’s limbs. The details are shown in Table 1. This patient showed extremely low levels of 17α-hydroxylated steroids (i.e., androgens, estrogen, cortisol, 11-deoxycortisol, and 17-OPH), increased levels of progesterone, and decreased levels of 17α hydroxyprogesterone and dehydroepiandrosterone sulfate. The patient did not receive any hormone therapy before admission. Although hypokalemia was present, the symptoms of fatigue were not obvious.

**Table 1:** Laboratory tests

Routine blood test		No obvious abnormality			
Routine urine test		Protein, leucocyte (−) Occult blood 2+ pH: 7.5 Specific gravity: 1.010			
Routine stool test + OB		No obvious abnormality.			
Biochemistry (blood, urine)		Blood electrolytes: blood sodium: 69 mmol/L, blood potassium: 1.5 mmol/L↓, blood chlorine: 98.13 mmol/L, blood calcium: 1.93 mmol/L↓, blood phosphorus: 0.99 mmol/L, blood magnesium: 0.62 mmol/L ↓, blood iron: 10.98 mmol/L; and bicarbonate: 34.56 mmol/L ↑.			
		Liver function, kidney function, blood glucose, blood lipid: normal			
		Urinary electrolytes: 24 hour urinary calcium: 5.64 mmol/day and 24 hour urinary magnesium: 5.19 mmol/day.			
Blood gas analysis		pH: 7.531 ↑, partial pressure of carbon dioxide: 32.10 mmHg ↓, partial pressure of oxygen: 98.90 mmHg, sodium ion: 139.00 mmol/L, potassium ion: 3.00 mmol/L ↓, ionic calcium: 1.06 mmol/L ↓, lactic acid: 1.30, standard bicarbonate: 28.50, P50 (act), Te: 23.04 mmHg, actual alkali residue: 4.60 mmol/L, and chloride ion: 105. 00 mmol/L.			
Thyroid function (blood)		TSH: 1.92 mIU/L, FT3: 5.69 pmol/L, and FT4: 14.38 pmol/L.			
A complete set of sex hormones (blood)		Estradiol: 28.00 pg/mL ↓, follicle stimulating hormone: 48.38 mIU/mL, luteinizing hormone: 20.29 mIU/mL, prolactin: 13.09 ng/mL, progesterone: 23.19 ng/mL, and testosterone: 0.37 ng/mL.			
Growth hormone (blood)		0.25 ng/mL			
Coagulation function (blood)		No obvious abnormality			
Glycated hemoglobin (blood)		5.6%			
Others		Dehydroepiandrosterone sulfate (blood): 22.6 μg/dL↓; 17α-hydroxyprogesterone (blood): 0.7 nmol/L↓; 17 ketosterol (17-KS) (urine): < 2.0 mg/24 h↓; 17 hydroxysteroid (17-OH) (urine): 9.6 mg/24 h			
24-hour urine potassium and synchronous blood potassium		Blood potassium (mmol/L)			24 hour urinary potassium (mmol/day)
	2018-04-12	2.94↓			106.82↑
	2018-04-13	3.10↓			96.72 ↑
Adrenocortical hormone, cortisol (blood)		8:00	16:00	24:00	
	ACTH (reference standard 1.6–13.9 pmol/L)	5.16	2.75	2.43	
	Cortisol (reference standard 185–624 nmol/L)	75.71↓	61.51↓	28.05↓	

	Lying position	Standing position
Renin activity (PRA) reference standard	0.78	0.57↓
(Lying position 0.15–2.33 ng/mL/h)		
(Standing position 1.31–3.95 ng/mL/h)		
Aldosterone (ALD) reference standard	410.01↑	416.96↑
(Lying position 10.00–160.00 pg/mL)		
(Standing position 40.00–310.00 pg/mL)		
ARR (ratio)	52.57↑	73.15↑

### 2.3 Imaging results

**Figure 2:**
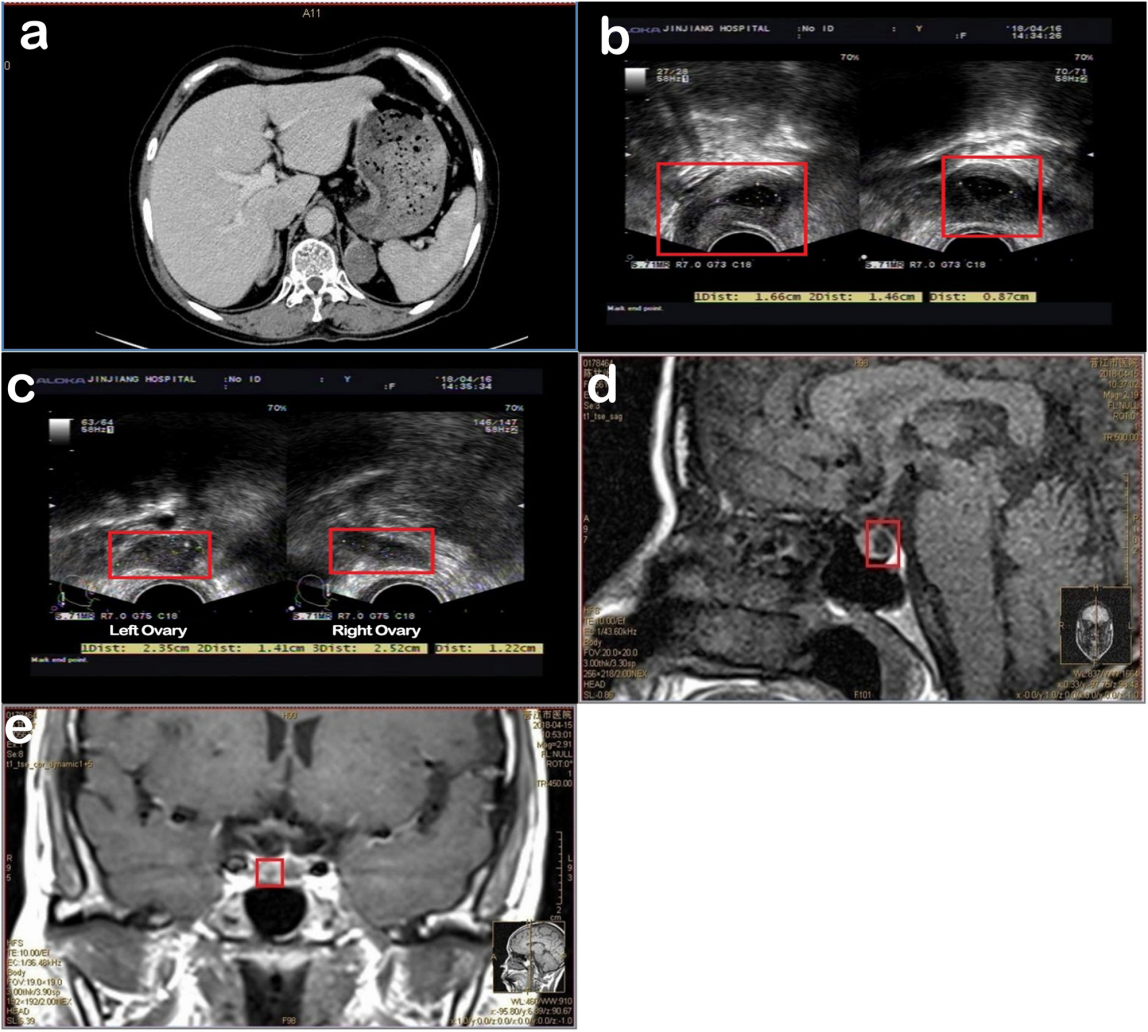
(a) Non-enhanced and enhanced CT scanning of adrenal gland; (b) transvaginal ultrasound of the female reproductive system 1, the female reproductive system was examined using transvaginal ultrasonography, and the results showed that the uterus was small (2.6 cm × 2.6 cm × 2.1 cm) and that the shape was regular; (c) transvaginal ultrasound of the female reproductive system 2, both the ovaries were small, the size of the left ovary was c. 2.5 cm × 1.2 cm, and the size of the right ovary was c. 2.4 cm × 1.4 cm. This needs to be differentiated from the ovaries of postmenopausal women because the vulva of this patient also shows undeveloped performance, which is different from the vulva of postmenopausal women, the reason for the small ovaries of this patient might be caused by pathological factors. (d) MRI1 of pituitary gland; (e) MRI 2 of pituitary gland.

Imaging data: a non-enhanced + enhanced computed tomography scan of the patient’s adrenal gland displayed multiple adenomas on both sides. The largest slice (c. 2.9 cm × 2.4 cm) was located on the left side (image in Figure 2a). The female reproductive system was examined using transvaginal ultrasonography, and the results showed that the uterus was small (2.6 cm × 2.6 cm × 2.1 cm) and that the shape was regular (image in Figure 2b). Both ovaries were small; the size of the left ovary was c. 2.5 cm × 1.2 cm, and the size of the right ovary was c. 2.4 cm × 1.4 cm (Figure 2c). The pituitary magnetic resonance imaging results showed an abnormal signal considered to be Rathke’s cyst at the posterior lobe junction (Figure 2d). Abnormal enhancement, which might refer to pituitary microadenoma, was further found on the right side of pituitary gland (Figure 2e). The gene detection and the karyotype results are shown in Table 1 and Figure 1b–e. The laboratory test results are shown in Table 2.

**Table 2:** Genetic testing

Experimental result statistics		Description of variant information					
Locus information	SNP locus base	Peak locus	Reference sequence base	Variant locus region sequence	Nucleotide changes	Amino acid changes	Variant type
CYP17A1-E1	TT	181	C	TCCCCAGACA[T/T]GGCCATATGC	c.138C > T	p.His46His	Synonymous variant
	TT	238	G	CCATCTATTC[T/T]GTTCGTATGG	c.195G > T	p.Ser65Ser	Synonymous variant
CYP17A1-E2	No variant						
CYP17A1-E3	No variant						
CYP17A1-E4	No variant						
CYP17A1-E5	No variant						
CYP17A1-E6	No variant						
CYP17A1-E7	GG	260	C	TGGCACCAGC[G/G]GGATCAGTTC	c.1226C > G	p.Pro409Arg	Missense variant
CYP17A1-E8	No variant						

After excluding other possible causes, hypokalemia was considered the cause of fatigue. After administration of a potassium supplement (oral and intravenous potassium supplement, 5–6 g/day for 2 weeks), the hypokalemia was not corrected. Clinical manifestations included chronic refractory hypokalemia, a high urinary potassium level, hypertension, female sexual infantilism (a small uterus, small ovaries, and immature external genitalia), primary amenorrhea, and infertility. The 24 h urine electrolytes indicated renal potassium loss, and the laboratory tests revealed a high serum aldosterone level, low serum renin activity, high ARR level, low serum cortisol, a non-feedback ACTH level increase, and sex hormones are at menopausal levels (low estrogen, high follicle stimulating hormone, and high luteinizing hormone). After potassium supplement, the blood calcium and magnesium levels were normal, and the patient’s urine calcium level was no longer low. Therefore, Gitelman syndrome and other diseases could be excluded. After the patient was treated with oral prednisone acetate tablets (5 mg in the morning and 2.5 mg in the evening), the blood potassium returned to and maintained at the normal level during follow-up.

The imaging findings showed a small uterus, multiple adrenal adenomas, and pituitary microadenomas. The above-listed evidence strongly suggests that the patient had congenital adrenal hyperplasia (CAH), a 17α-hydroxylase deficiency condition (OMIM number #202110, https://www.omim.org/entry/202110? search = %23202110&highlight = 202110#mimProteinLinksFold). Furthermore, the decreases in dehydroepiandrosterone sulfate and 17-ketosterol levels further supported 17α-hydroxylase deletion. Genetic testing revealed that the cytosine (nucleotide C) at the site 1226 of the 17α-hydroxylase-related CYP17 gene underwent a missense pathogenic variant and was changed to guanine (nucleotide G). Next it was missense mutated to arginine (Arg), which may have affected the protein conformation, by the amino acid proline (Pro) at the 409 position in the protein. Eventually, the 17α-hydroxylase function became abnormal. According to the patient’s sex-related clinical manifestations and karyotype results, the diagnosis was determined as complicated triple X syndrome (47, XXX [3]/46, XX [47]). She was not a pure 47,XXX karyotype, but a 47,XXX [3]/46,XX [47] low level chimeric karyotype, with insignificant intellectual effects, self-care, no speech impairment, and no signs of microcephaly (no intelligence assessment was performed).

### 2.4 Genetic testing results

Direct DNA sequencing, which was performed in a third-party institution [12], revealed that this patient had a homozygous change within gene CYP17A1. The pathogenic variant is a missense pathogenic variant, g.5582C. G, (5), located in exon 7 (equivalent to nucleotide position 7,450 in the GenBank sequence with Accession Number M19489), changing the codon 409 from CCG to CGG, and changing the coded amino acid from pro-line to arginine, i.e., P409R.

## 3 Discussion

This patient was characterized by intractable hypokalemia, sexual hypoplasia, infertility, low serum cortisol, high serum aldosterone, low serum renin, low 17-OPH, and decreased sex hormone levels. This was accompanied by hypertension, which is related to high aldosterone levels. She was admitted to the hospital due to hypokalemia. The examinations revealed abnormal mineralocorticoids (i.e., high blood aldosterone, low blood renin). The main physiological role of mineralocorticoids is to promote the reabsorption of sodium in the renal tubules while retaining water and excreting potassium. It coordinates with the antidiuretic hormone secreted by the hypothalamus to maintain the balance of water and electrolytes in the body. Aldosterone is one of the common and the most powerful mineralocorticoid. The daily secretion of aldosterone is usually very small, such as excessive secretion of aldosterone due to certain circumstances, its significant sodium water retention, and potassium excretion effects can cause hypokalemia, tissue edema, and hypertension. If the level of mineralocorticoid secretion is too low, it causes water and sodium loss and decrease in blood pressure. She developed hypokalemia due to renal potassium loss due to elevated aldosterone levels, accompanied by hypertension. The imaging findings indicated the presence of multiple bilateral adrenal gland adenomas, consistent with the manifestations of 17α-hydroxylase deficiency. The gene sequencing results showed that the nucleotide C at site 1,226 of the CYP17 gene was missense mutated to the nucleotide G, and that, finally, Pro (the 409th amino acid in the protein) was missense mutated to Arg. This affected the protein conformation and finally resulted in a 17α-hydroxylase deficiency dysfunction. Therefore, a gene-level diagnosis could be made. This condition may be caused by consanguineous marriage. A study from Brazil showed that patients with 17α-hydroxylase deficiency have a high rate of misdiagnosis [13]. The most common misdiagnoses included essential hypertension (55%), simple gonadal dysplasia (35%), and androgen resistance syndrome (21%). The median age at first diagnosis and diagnosis were 13.2 and 16.5 years, respectively, and the mean time between diagnosis was 3.2 years [13]. A few previous studies have reported the specific pathogenic variant in CYP17A1-E7 [14,15]. The study by Lam et al. reported a patient in Thailand and suggested that g.6333--6341delGACTCTTTCA may be a prevalent pathogenic variant causing P450c17 deficiency in Southeast Asia [16].

The imaging data showed that both ovaries of the patient were small. However, it was different from the ovaries of postmenopausal women. The vulva of this patient also exhibited undeveloped performance, which is different from the vulva of postmenopausal women. The reason why this patient had small ovaries might be due to pathological factors.

**Figure 3:**
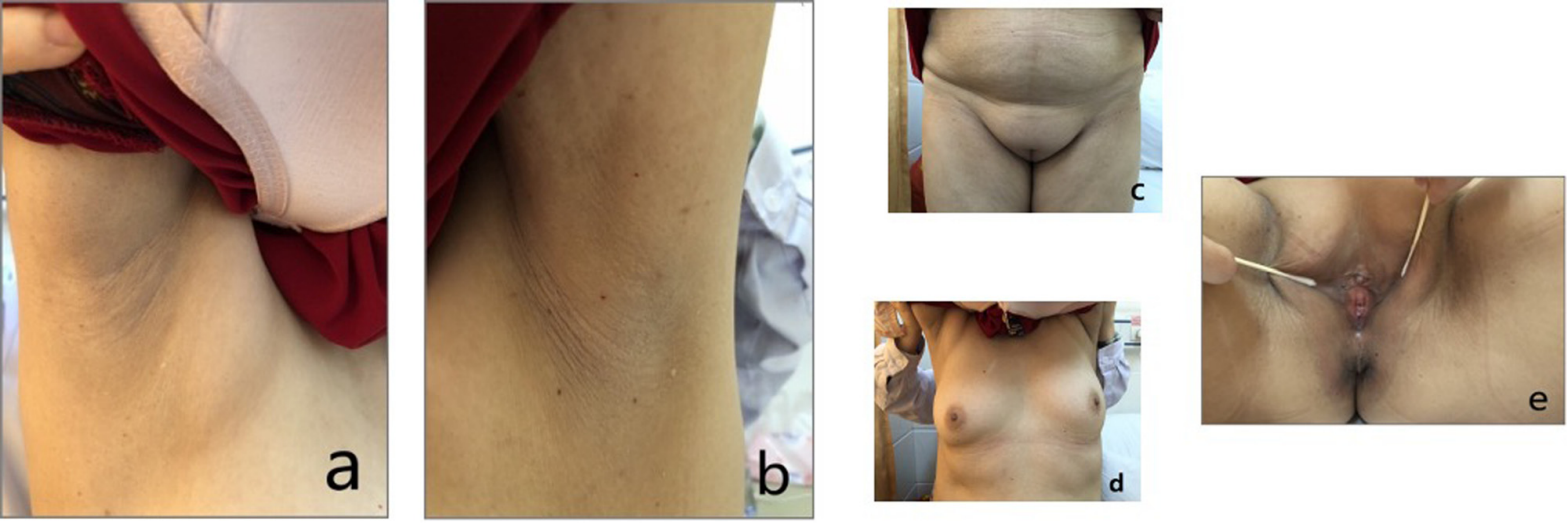
(a) No axillary hair in right armpit; (b) no axillary hair in left armpit; (c) no perineal pubic hair; (d) the areola is light brown; (e) hypoplasia of external genitalia (sexual infantility of labia major, labia minor, and clitoris).

A 17α-hydroxylase deficiency is a hereditary autosomal recessive disease. It is a very rare type of CAH accounting for only 1% and with a clinical prevalence rate of 1/50,000 [1]. Patients with triple X syndrome are even rarer, and at present no relevant pieces of literature include reports of a 17α-hydroxylase deficiency complicated with triple X syndrome. To our knowledge, this is the first reported case of the particular combination worldwide. It is unclear whether there are certain factors and degrees of interaction between the 17α-hydroxylase deficiency and triple X syndrome in the progress of the disease. Triple X syndrome is a common abnormal sex chromosome syndrome in women, with a 0.73–1.00/1,000 incidence [17,18]. It was first described in 1959 by Jacobs et al. and called super-female syndrome (corresponding to 47, XYY super-male syndrome). Most patients do not have an abnormal phenotype. A small number of cases had developmental delays or psychological disorders [19,20]. However, they may sometimes experience complications, such as certain mild birth defects (e.g., wide eye distance, wide breast distance, short head deformity, and microcephalus) [21]. The sterility incidence is slightly higher in females with triple X syndrome than in the general population. “Super-female” patients are usually tall [22] and may have decreased menstruation, secondary amenorrhea, or premature ovarian failure. The height of the present patient was 168 cm, which was slightly greater than the height of the patient’s female relatives of the same age. The patient’s breast distance was also slightly wider (Figure 3), and infertility with amenorrhea were consistent with triple X syndrome characteristics. The karyotype revealed that the patient was a 47, XXX [3]/46, XX [47] low-level chimeric karyotype. In this case, the patient still had a normal female appearance, breast development, and no vaginal malformation. The patient had intercourse with her husband despite the 17α-hydroxylase deficiency resulting in low estrogen levels. The 17α-hydroxylase gene abnormality leads to a decrease in estrogen levels. Theoretically, it should have the characteristics of non-female appearance and decreased libido, but the patient also has three X syndromes, which makes the patient have the characteristics of female appearance, breast development, and sexual desire, which is also a manifestation of atypical manifestations of the disease. It has been indirectly proven that the concomitant triple X syndrome has a super-female effect.

On admission, the patient’s cortisol level was decreased; however, the ACTH level presented a non-feedback increase. This was analyzed as follows: because the disease could not be diagnosed at the beginning, it may be related to different manifestations at different stages of the disease. The reason may be that because of the long disease history, the ACTH level increased in the early stage of the disease, leading to adrenal cortex hyperplasia. However, with time and disease development, the corticosterone level increased in the later stage, and a very high level of corticosterone may have replaced the role of cortisol and further inhibited ACTH secretion, so that the ACTH level was not high. The adrenal imaging findings of patients with CAH are mainly bilateral adrenal hyperplasia or nodular thickening manifesting as adenoma formation. This has been reported; however, it is rare. Nagai et al. [23] reported a case of 17α-hydroxylase deficiency with left adrenal myelolipoma. In this case, there were no significant changes in BP, serum potassium, and hormone levels after tumor resection, suggesting that it was a non-functional tumor. However, the difference between ACTH-involved adrenal hyperplasia and adenoma formation is still unclear, which is worth further analysis. Fertility problem is another issue for patients with 17α-hydroxylase deficiency. Previous studies have reported cases who were conceived by *in vitro* fertilization and had successful live birth [24,25], suggesting that patients with CYP17A1 gene pathogenic variant may achieve fertility through reproductive technology.

## 4 Conclusion

The patient presented in this study is (to our knowledge) the first reported case of CAH caused by 17α-hydroxylase deficiency (c.1226C > G) and triple X syndrome worldwide. The uniqueness of this case is that this patient has two very rare genetic diseases, probably due to the marriage of parents between close relatives. The 7α-hydroxylase gene causes the patient to have high blood aldosterone and low blood cortisol and estrogen, and then show the characteristics of hypokalemia, hypertension, primary amenorrhea, infertility, etc. However, the combined triple X syndrome makes this patient have a female appearance, breast development, and sexual desire. The challenge of diagnosis is that, for patients with CAH, the usual focus is on the detection of related enzyme genes, and less attention is paid to chromosomal abnormalities. The offspring of CAH may have abnormalities at the genetic and chromosomal levels. This case report may provide some references for clinicians.
